# Structure and Rheological Properties of Bovine Aortic Heart Valve and Pericardium Tissue: Implications in Bioprosthetic and Tissue-Engineered Heart Valves

**DOI:** 10.1155/2019/3290370

**Published:** 2019-12-28

**Authors:** Hani A. Alhadrami, Raza ur Rehman Syed, Alap Ali Zahid, Rashid Ahmed, Shajia Hasan, Anwarul Hasan

**Affiliations:** ^1^Faculty of Applied Medical Sciences, Department of Medical Laboratory Technology, King Abdulaziz University, P.O. Box 80402, Jeddah 21589, Saudi Arabia; ^2^King Fahd Medical Research Centre, King Abdulaziz University, P.O. Box 80402, Jeddah 21589, Saudi Arabia; ^3^Department of Mechanical and Industrial Engineering, College of Engineering, Qatar University, Doha 2713, Qatar; ^4^Biomedical Research Center, Qatar University, Doha 2713, Qatar; ^5^College of Medicine, Qatar University, 2713 Doha, Qatar

## Abstract

Heart valve (HV) diseases are among the leading causes of cardiac failure and deaths. Of the various HV diseases, damaged HV leaflets are among the primary culprits. In many cases, impaired HV restoration is not always possible, and the replacement of valves becomes necessary. Bioprosthetic HVs have been used for the replacement of the diseased valves, which is obtained from the sources of bovine and porcine origin, while tissue-engineered heart valves (TEHV) have emerged as a promising future solution. The bioprosthetic valves are prone to become calcified, and thus they last for only ten to fifteen years. The adequate understanding of the correlations between the biomechanics and rheological properties of native HV tissues can enable us to improve the durability of the bioprosthetic HV as well as help in the development of tissue-engineered heart valves (TEHV). In this study, the structural and rheological properties of native bovine aortic HV and pericardium tissues were investigated. The microstructures of the tissues were investigated using scanning electron microscopy, while the rheological properties were studied using oscillatory shear measurement and creep test. The reported results provide significant insights into the correlations between the microstructure and viscoelastic properties of the bovine aortic HV and pericardium tissues.

## 1. Introduction

Heart valve (HV) diseases are among the major causes of death among elderly people worldwide [1]. Every year, around 30,000 people around the world undergo HV replacement surgeries, while just in 2013, around 275,000 people died worldwide due to various HV diseases [2–4]. Among various HV diseases, aortic valve (AV) disease is the third most common valvular disease causing a large number of deaths every year [5]. According to one estimate, the prevalence of HV replacement surgery is going to be increased from 290,000 to 890,000 between 2003 and 2050 [6, 7]. Most HV diseases are treated by repairing the damaged valve leaflets. However, the grafting of the valves sometimes becomes challenging and not feasible; therefore, the replacement or surgery of HV becomes necessary [4].

There are two types of valves available for the replacement of damaged HVs: biological valves and mechanical valves [8, 9]. Biological valves are obtained from the bovine or porcine origin, which sometimes may get rejected by the human immune system [10–12]. These bioprosthetic HVs are prone to calcification which causes the valve to become stiffer and thicker [13] and leads to incomplete opening and closing of the valve resulting in backflow and leaks during the normal function [10]. Besides, it requires replacement after approximately 10 to 15 years [14]. Mechanical valves, on the contrary, are susceptible to inflammation and thrombosis [15] and require the anticoagulation drug to prevent thrombosis.

Tissue engineering offers a viable solution for the fabrication of artificial biomimetic HVs [4, 16–18]. Various biodegradable polymeric materials have been employed to develop biodegradable scaffolds which can be seeded with HV cells to develop HV tissue [19, 20]. The cell-seeded constructs can then be implanted into a patient's heart. Over the time, the scaffold biodegrades, while the HV cells create new extracellular matrices and structure themselves to provide normal tissue function [21–25]. One of the major challenges in the development of tissue-engineered heart valves (TEHV) and its successful clinical application is the obscure understanding of the structure to property correlations of HV tissues. Further, the prospect of TEHV is highly reliant on the ability to mimic the properties and functionalities of the native HV prior to implantation. For TEHV, it is crucial to provide enough mechanical strength and viscoelastic properties to hold the hemodynamic pressures and the cardiac cycles [26–30]. Hence, in order to develop the TEHV, it is necessary to understand the properties of different native HVs. Several studies have been performed on the biomechanical properties of HV leaflets, where the focus has mostly been on the mechanical perspective of the HV tissues [11, 31]. The need for better understanding and more effective characterization of viscoelastic properties of HV tissues is required. Furthermore, there is no systemic study found for the rheological properties of bovine aortic heart valves (AHV) and pericardium tissues. The proper understanding of the rheological properties of native HV tissues can pave the way for improving the behavior and properties of implantable TEHVs as well as it may help in enhancing the durability of bioprosthetic HVs prior to the implantation.

In this study, the rheological properties of bovine AHV tissues and pericardium tissues have been investigated to understand the viscoelastic behavior of HVs and pericardium tissues. In addition, the effect of the paraformaldehyde fixation on AHV and pericardium tissue was studied. Our experimental research includes the use of an advanced dynamic shear rheometer to investigate the rheological behavior of native bovine aortic HV and pericardium tissues which will facilitate the pathway for development of new generation tissue-engineered heart valve devices. This study provides significant information which can be useful in enhancing the properties of bioprosthetic bovine HV as well as help in the development of implantable TEHVs.

## 2. Materials and Methods

### 2.1. Source of Bovine AHV and Pericardium Tissue

**Figure 1:**
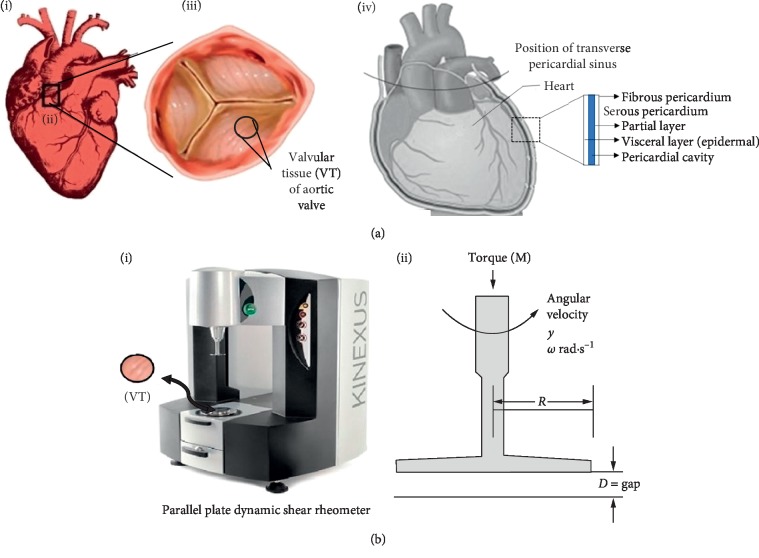
Schematic representation of the extraction of the aortic heart valve from bovine heart and the uniaxial testing. (a) (i) indicates the location of the bovine heart, (ii) indicates the location of the aortic valve in the heart, (iii) indicates the three leaflets of the aortic valve with the representation of the dissection of the valvular tissue, and (iv) indicates position and layers of pericardium. (b) (i) indicates the parallel plate rheometer; the VT of 8 mm is placed between the plates of rheometer to study the viscoelastic properties, and (ii) indiactes the characteristics and phenomena in action during a rheometric experiment.

Fresh bovine (cow) heart and pericardium tissues were obtained from the local slaughterhouse in Doha, Qatar. The pericardium tissue was separated from the heart followed by the dissection of the heart and the extraction of the AHV leaflets. The obtained AHV leaflets and pericardium tissue were washed with the sterile PBS and kept in PBS in two separate Petri dishes. Specimens of 8 mm diameter were punched from tissues using a biopsy punch. For the aortic valve leaflets, the samples were punched from the belly region, which is the main part of each leaflet [32], whereas for the pericardium tissue, the samples were punched from the middle region to ensure uniform thickness. A detailed schematic of the bovine HV extraction and adopted procedure has been shown Figure 1.

### 2.2. Fixation in Formaldehyde

The bovine AHV leaflets and the pericardium tissue were divided into two groups, where one group of samples was stored in PBS and the other group was fixed in 4% paraformaldehyde solution. Formaldehyde fixations lower the antigenicity and stabilize the tissue against proteolytic degradation. Eight samples of AHV tissues and pericardium tissues were fixed with formaldehyde, and four samples were preserved in PBS for a different period of times as necessary.

### 2.3. Scanning Electron Microscopy (SEM)

The microstructure of the AHV tissue and pericardium tissue were investigated by using the SEM. The samples were cut using a 8 mm biopsy punch as mentioned earlier. Subsequently, samples were washed with distilled water and then dehydrated with graded ethanol of 70%, 85%, 95%, and 100%. Following this, samples were air-dried at room temperature under the hood and placed into the studs using the conducting tape, and then sputter coating was performed with a thin layer of gold of about 200–500 Å. Finally, samples were added into the SEM vacuum chamber, and images were taken under an accelerating voltage of 30 kV using a secondary electron detector at different magnifications.

### 2.4. Rheological Properties

The bovine HV and pericardium tissues were subjected to amplitude sweep, frequency sweep, and creep test using a Malvern Kinexus dynamic shear rheometer. Frequency sweep test was carried out by applying strain at 100% with the frequency ranging from 0 to 100 rad/sec. The oscillatory shear properties in terms of modulus of elasticity, storage modulus, loss modulus, complex modulus, complex viscosity, and creep recovery properties were thoroughly investigated. For the frequency sweep test, the oscillation frequency was varied from 0.001 to 10 Hz.

The equations used to calculate the shear rate and shear stress from the rheometer data were as follows:

(1) $$\begin{array}{c} \text{shear rate}\left({\text{s}}^{-1}\right)=F\dot{\gamma }\omega ,\\ \text{shear stress}\left(\text{Pa}\right)=F\sigma T, \end{array}$$

where $F\dot{\gamma }=R/D$ and *F*_*σ*_=2/(*πR*^3^); *F*: force (N); *ω*: angular velocity (rad/sec); *T*: torque (N.m); *R*: radius of the rheometer plate (m); and *D*: gap distance between rheometer plates (m).

The creep test was performed at 0.5 kPa and 5 kPa, for 300 s in the linear viscoelasticity range. The stress was then removed, and the sample strain percentage was recorded for further 300 s during the recovery stage.

## 3. Results and Discussion

### 3.1. Scanning Electron Microscopy

**Figure 2:**
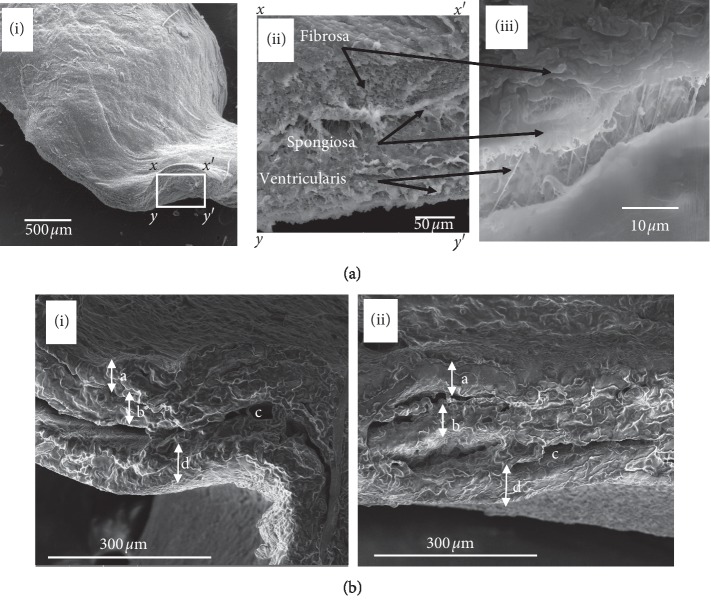
(a) SEM of a cross section of the aortic bovine heart valve leaflets. (i) indicates a cross section of the heart valve at magnification 54x; (ii) and (iii) indicate the three distinct layers fibrosa, spongiosa, and ventricularis clearly visible at magnification of 461x and 3350x, respectively. (b) SEM of cross section of the bovine pericardium: (i) and (ii) indicate two layers of pericardium; a and b represent serous pericardium of 55.85 and 55.95 thickness (partial layer and visceral layer/epidermal), (c) represents pericardial cavity, and (d) represents fibrous pericardium of 55.85 *μ*m thickness.

The SEM cross-section images of the bovine AHV tissue and pericardium tissue are shown in Figure 2. Three distinct layers of the aortic valve fibrosa, spongiosa, and ventricularis can be seen in Figure 2(a) ((i), (ii), and (iii)). These three layers are mainly composed of collagen, glycosaminoglycan (GAGs), and elastin, respectively.  Figure 2(b) shows the SEM cross-section image of the bovine pericardium tissue. The two layers of pericardium, fibrous pericardium, and serous pericardium (partial layer and visceral layer/epidermal) and the space between two layers of the serous pericardium (pericardial cavity) can be seen in Figure 2(b) ((i) and (ii)).

The SEM images confirm the layers of HV tissue and the pericardium tissue. Mechanical stiffness and stability are attributed to the fibrosa and ventricularis layers due to their high composition of collagen fibres [32]. The fibrosa layer is close to the outflow region (the pulmonary arteries or the aorta) and composed of circumferentially aligned collagen fibres. This structural layer has a potential to provide tensile strength to the valve leaflet during its opening [2]. The ventricularis is closer to the ventricular area and composed of radially aligned elastin fibres dispersed in a collagen matrix. This gives the ventricularis the ability to extend and recoil during the diastole and systole of the cardiac cycle. However, the pericardium consists of only two layers (fibrous and serous layers). The serous fluid in the pericardial cavity (the space between two layers of serous pericardium) protects the heart from any kind of jerk or shock.

### 3.2. Viscoelastic Properties

#### 3.2.1. Analysis of Frequency Sweep Test

**Figure 3:**
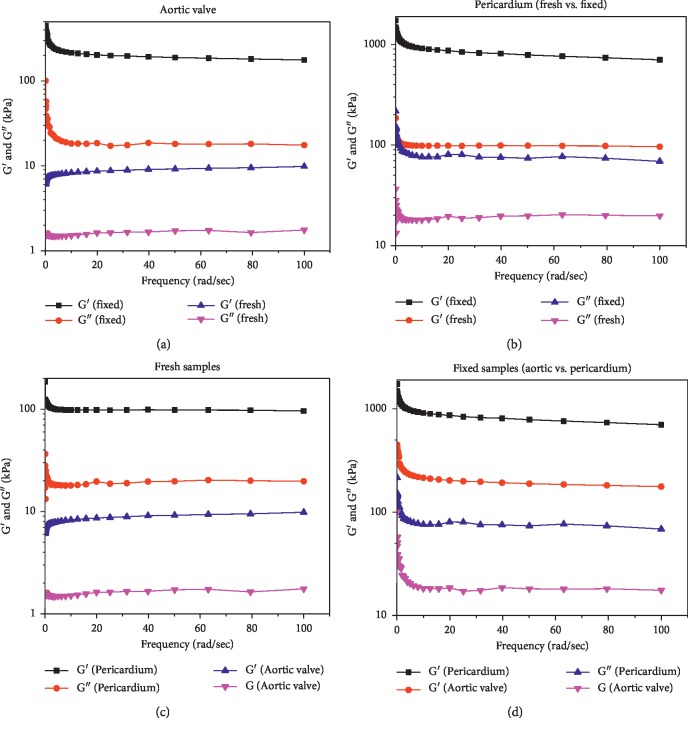
(a) Storage and loss moduli curve for fresh and fixed samples of bovine aortic valve. Mean storage modulus ± S.E. The two curves are significantly different with *P* value <0.05. (b) Storage and loss moduli curve for fresh and fixed samples of bovine pericardium tissues. Comparison of storage and loss modulus curves between (c) fresh samples and (d) fixed samples of aortic valve and pericardium tissues.

The storage and loss moduli of fresh and fixed samples of the AHV tissue and pericardium tissue are shown in Figure 3.

The result depicts that the valves exhibit a solid/rigid structure. The storage modulus (G′) of the AHV tissue fixed with formaldehyde at an average of three different frequencies (1, 10, and 100 rad/sec) was 28 times higher compared with the fresh samples. However, the loss moduli of the samples fixed with formaldehyde was 14 times higher than those for the fresh counterpart. Moreover, the storage and complex moduli of pericardium tissue were much higher compared with the AHV tissue. The ranges of storage and loss modulus for the fixed samples of pericardium were about 4 times higher compared with those of fixed HV tissues. However, for the fresh samples, the ranges of pericardium were about 12 times higher compared with those of AHV tissues. The higher values of the fixed samples can be attributed to the fact that the formaldehyde fixation lowers the antigenicity and stabilize the tissue against proteolytic degradation.

#### 3.2.2. Analysis of Complex Viscosity and Complex Modulus

**Figure 4:**
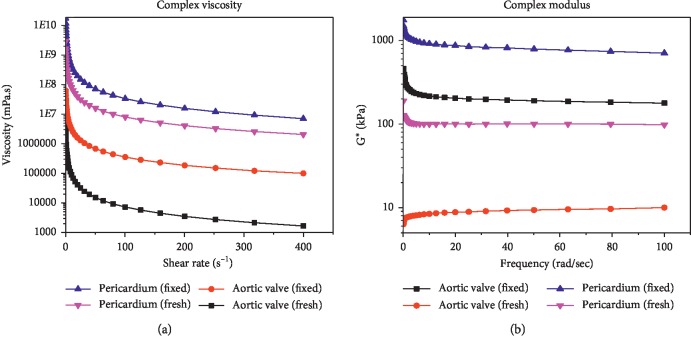
(a) Complex viscosity comparison b/w fixed and fresh samples of aortic heart valve and pericardium tissue. (b) Frequency dependent complex modulus (G∗): samples of aortic valve and pericardium tissues fixed with formaldehyde and fresh samples. Mean complex modulus ± SE.

Complex viscosities for the AHV tissue and the pericardium tissue at a strain rate of 5% are shown in Figure 4. The viscosities of all samples decreased with the increase of shear rate, which confirms the shear thinning behavior of the tissues. Moreover, the viscosity of fixed samples compared with the fresh samples and the viscosity of pericardium tissue compared with the AHV tissue was significantly higher (one order of magnitude higher at high frequencies) which proves that the fixation of the valves with formaldehyde altered the viscoelastic properties of the valves. At a shear rate of 100·s^−1^, the viscosity of the fixed samples of pericardium tissues was 4 times higher compared with that of the fresh samples, and the viscosity of fixed sample of AHV tissue was 49 times higher compared with fresh counterpart. Viscosity of the fixed samples of pericardium was comparatively 95 times higher and those of the fresh were 1100 times higher than the AHV tissues. Furthermore, the complex modulus of pericardium tissue was much higher compared with the AHV tissue.

#### 3.2.3. Analysis of Creep Recovery Test

**Figure 5:**
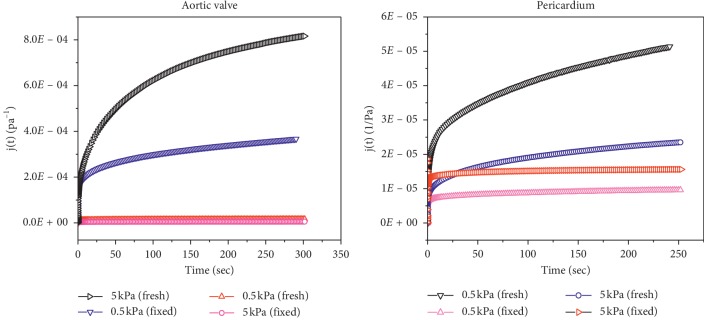
Analysis of creep recovery test applying stress at 5 kPa and 0.5 kPa on the samples of bovine aortic valve and pericardium tissue. The *Y* axis shows the creep compliance valve with respect to time. The creep compliance of fresh sample for both the stresses at 0.5 kPa and 5 kPa is much higher than the fixed samples of bovine aortic heart valve and pericardium tissue at stresses of 0.5 and 5 kPa.

The stress-dependence of recoverable compliance for the fresh and formaldehyde-fixed AHV and pericardium tissue are represented in Figure 5. The behavior is linear for both stresses, namely, 0.5 kPa and 5 kPa. Recovery compliance was higher at lower stress compared with that at the higher one. Through the creep tests, it was clear that the fixation of the HVs and pericardium tissue did affect the viscoelasticity and rheological properties of the HVs. By fixing the samples, the reaction of the samples was reduced, i.e., they became stiffer when they were fixed with formaldehyde. The reactions were less by a whole order of magnitude. The effect of time did not pose much influence on the overall experiment. However, by fixing the sample in formaldehyde, it was observed that the fixed AHV tissues and pericardium tissue had much higher strength compared with those of the fresh sample. Another proof of how the formaldehyde fixation drastically affected the stiffness of the valve was in the fact that the extension of the formaldehyde-fixed samples at the stress of 5 kPa was less than that of the fresh samples at 0.5 kPa.

Thus, the experimental results discussed above reveal that the highest value for the viscoelastic properties were obtained for the formaldehyde-fixed pericardium tissues. This justifies the fact that in clinical practice, the bioprosthetic HV made from the bovine pericardium tissue instead of the bovine AHV leaflets. In addition, the current study also shows that the properties of the biological tissue implants can be significantly improved by proper fixation.

## 4. Discussions

In this study, we have carried out a detailed study on the structure and rheological properties of bovine AHV tissue and pericardium tissue: implications in bioprosthetic and TEHVs. Cardiovascular diseases have emerged as one of main challenge these days as the number of patients suffering from these diseases have increased manifold over the last one century. Especially, enormous increase in HVs diseases has adversely affected the activities of humans, and there is growing demand to find safer techniques for the replacement of damaged HVs. Meanwhile, the etiology of the disease has been changed greatly due to tissue damages taking place in HVs leading to a shift from rheumatic to degenerative disease. This has greatly changed the characteristics of HV diseases due to wide distribution of lesions in the tissues of HVs. Replacing the HVs with prosthetic HVs or cryopreserved homograft valves offers best solution for highly damaged HVs. However, short-term durability, limited supply of cryopreserved homografts, and continuous need of anticoagulation drugs are some of impediments for successful transplantation of bioprosthetic valves [33]. Furthermore, a series of surgeries are often required for replacing pediatric and young adult HVs as the implants often lack compatibility with host tissue in the form of mechanical properties leading to stunted growth. TEHV offer best solution for replacing the damaged HVs in cases where a multiple set of surgeries are needed for replacement of impaired HVs. Deficiencies of prosthetic HVs transplants can be overcome by designing a TEHV that has nonthrombogenic potential. Besides, the use of minimally invasive cell sources from autologous tissues composed of smooth muscle progenitor cells [34], bone marrow-derived MSCs [35], circulating progenitor cells such as EPCs [36], adipose-derived MSCs [37], and induced pluripotent stem cells [38] exert beneficial effects on TEHV. Viscoelastic properties of host tissue play a significant role in success of HVs transplants as it is seen that shrinkage of HVs leaflets leads to increased expression of contractile proteins of the transplants and leads to tissue rejection [35, 39].

Therefore, during present work, the layers of AHV tissue and pericardium tissue were confirmed by SEM images. The viscoelastic properties of AHV and pericardium tissues were examined and quantified by using an advanced rheometer (Malvern Kinexus dynamic shear rheometer). In one study, the effect of hyaluronidase was tested on tensile and mechanical properties of AHV using uniaxial mechanical tester. They showed that there was significant change in elastic modulus, the maximum stress, and hysteresis of AHV treated with hyaluronidase due to digestion of glycosaminoglycans [40]. In another study, a relationship between mechanical properties and histology of porcine atrioventricular HVs was determined using biaxial mechanical tester. The results showed that there was continuous increase in stiffening of HV tissues with the increase in loading rate and temperature in atrioventricular HV leaflets. There was similar amount of chordae quantities in the porcine mitral and tricuspid valves but relatively higher chordae in the porcine compared with the bovine valves [41]. The effect of formaldehyde fixation on the viscoelastic properties of AV and pericardium tissues was evaluated and compared with the fresh samples. The ranges of storage and loss moduli of formaldehyde-fixed samples for both AHV leaflets and pericardium tissue were significantly higher than the fresh samples in the frequency sweep test. The obtained results were comparable with the work reported by Anthony et al. performed on porcine AV xenograft. There was enhanced degree of cross-linking due to increased penetration of the glutaraldehyde fixative agent that leads to higher cross-linking of collagen free molecules. It was considered that improved stress relaxation potential was due to an increase in potential shearing forces among the adjacent collagen fibres that was one of reason which preserved the stress-reducing potential of the fresh and untreated valves [12]. Furthermore, the pericardium tissues have higher values of storage and loss moduli compared with its AHV counterparts. Also, the complex viscosity of fixed samples compared with the fresh samples and the viscosity of pericardium tissue compared with the AHV tissues tissue was significantly higher. Results altogether demonstrated that the fixation of AHV and pericardium tissues have enhanced the viscoelastic properties. The pericardium tissues have more mechanical strength than the AHV leaflets, which makes them more feasible for HV replacement. As Paola et al. report in his recent study that the bovine and porcine pericardium tissues are most preferable for their availability and traceability. On comparison with porcine pericardial tissue, bovine pericardium tissue is extensively studied due to its optimal mechanical strength for the development of bioprosthetic and TEHV [42]. The complex viscosities of all the samples have decreased with the increase of shear rate, which confirms the shear thinning behavior of the tissues. The creep recovery results depict that by fixing the samples, the reaction of the samples was reduced, i.e., they became stiffer when they were fixed with formaldehyde. The results obtained during present work will help in understanding the role of viscoelastic properties in developing new TEHVs, and our future studies will be focused in this direction. For example, we can use it for developing HVs with improved geometry, better HV design prepared from the cells of alternative sources and avoid the agents which inhibit the contraction of proteins by localized delivery of contractile proteins. The study will immensely help in finding a way for developing a TEHV that can be transplanted by minimally invasive way involving catheter-based delivery and transapical delivery methods [43]. The study will also amply help in designing and developing TEHV for applications in pediatric patients where immunogenic response fails to form clot and leads to tissue rejection. This will also help in fabrication of simpler but functional TEHV leaflets comparable with native three-layered HVs in the area of cardiovascular tissue engineering.

## 5. Conclusions

The high prevalence of HV surgeries necessitates a need to improve the properties of bioprosthetic HV implants and development of TEHV. Multiple investigations were performed in the past to determine the mechanical properties of native HVs which provided significant insights. However, the rheological properties or viscoelastic properties of HV tissues and pericardium tissues remained unexplored. In this study, the morphological and the rheological properties of the bovine AHV tissues and pericardium tissues have been investigated. The results depicted that the bovine AHV tissues and pericardium tissues exhibit unique and interesting viscoelastic behavior which give them the ability to go through millions of cycles of opening and closing during the cardiac cycle without any fatigue. The viscoelastic and creep behavior of the AHV and pericardium tissue improved significantly by fixation with the formaldehyde. These results will help in improving the bioprosthetic bovine HVs and in developing the improved TEHVs which could offer longer durability and less side effects. This will also facilitate the pathway for development of new generation tissue-engineered heart valve devices.

## Data Availability

All experiment related data and files used to support the findings of this study are available from the corresponding author upon request.
